# Supplementary material for: An Automated High-Accuracy Detection Scheme for Myocardial Ischemia Based on Multi-Lead Long-Interval ECG and Choi-Williams Time-Frequency Analysis Incorporating a Multi-Class SVM Classifier
Source: Sensors (Basel). 2021 Mar 26;21(7):2311. doi: 10.3390/s21072311 (PMC8037073; doi:10.3390/s21072311)
Supplement: Supplementary file 1 [file sensors-21-02311-s001.pdf]

Supplementary Materials: Table S1. Proposed scheme evaluation applied to European ST-T database, Table S2. Proposed scheme evaluation applied to MIT Fantasia database, and Table S3. Proposed scheme evaluation applied to collected ischemia database.

Table S1. Proposed scheme evaluation applied to European ST-T database

Records	Leads Group 1				Leads Group 2				Records	Leads Group 1				Leads Group 2			
	TP	TN	FP	FN	TP	TN	FP	FN		TP	TN	FP	FN	TP	TN	FP	FN
e0103	118	1	0	1	117	1	0	2	e0211	120	0	0	0	119	0	0	1
e0104	117	2	0	1	117	0	0	3	e0212	118	1	0	1	120	0	0	0
e0105	119	1	0	0	120	0	0	0	e0213	119	1	0	0	118	1	0	1
e0106	118	1	1	0	117	1	0	2	e0302	120	0	0	0	115	2	0	3
e0107	119	1	0	0	118	0	0	2	e0303	120	0	0	0	117	1	0	2
e0108	118	1	0	1	120	0	0	0	e0304	118	2	0	0	119	0	0	1
e0110	117	0	1	2	117	0	0	3	e0305	119	0	0	1	120	0	0	0
e0111	120	0	0	0	118	2	0	0	e0306	120	0	0	0	120	0	0	0
e0112	118	1	0	1	118	1	0	1	e0403	120	0	0	0	119	0	0	1
e0113	120	0	0	0	118	1	0	1	e0404	119	1	0	1	119	0	0	1
e0114	120	0	0	0	119	0	0	1	e0405	118	1	1	0	118	2	0	0
e0115	119	1	0	0	118	2	0	0	e0406	119	0	0	0	119	0	0	1
e0116	118	1	0	1	118	2	0	0	e0408	119	1	0	0	119	1	0	0
e0118	120	0	0	0	117	1	0	2	e0409	119	1	0	0	119	1	0	0
e0119	119	1	0	0	118	2	0	0	e0410	119	0	0	1	118	2	0	0
e0121	119	1	0	0	120	0	0	0	e0411	118	1	0	1	118	2	0	0
e0122	116	3	0	1	120	0	0	0	e0413	118	1	0	1	120	0	0	0
e0123	117	1	0	2	116	2	0	2	e0415	118	1	0	1	118	2	0	0
e0124	115	3	0	2	118	2	0	0	e0417	118	1	0	1	114	2	0	4
e0125	119	1	0	0	117	2	0	1	e0418	117	1	0	2	119	1	0	0
e0126	120	0	0	0	118	2	0	0	e0501	119	0	0	1	119	0	0	1
e0127	118	2	0	0	118	0	0	2	e0509	119	0	0	1	118	0	0	2
e0129	118	2	0	0	119	1	0	0	e0515	120	0	0	0	118	0	0	2
e0133	119	1	0	0	118	2	0	0	e0601	120	0	0	0	119	1	0	0
e0136	117	1	0	2	117	1	0	2	e0602	118	2	0	0	119	0	0	1
e0139	119	1	0	0	119	1	0	0	e0603	119	1	0	0	118	1	0	1
e0147	117	2	0	1	119	1	0	0	e0604	118	0	1	1	118	0	0	2
e0148	118	2	0	0	117	2	0	1	e0605	118	1	0	1	118	0	0	2
e0151	118	1	0	1	119	1	0	0	e0606	118	1	0	1	116	2	0	2
e0154	120	0	0	0	119	1	0	0	e0607	118	0	0	2	120	0	0	0
e0155	118	2	0	0	118	1	0	1	e0609	117	2	0	1	120	0	0	0
e0159	118	1	1	0	118	1	0	1	e0610	116	2	0	2	116	2	0	2
e0161	118	1	0	1	116	4	0	0	e0611	117	1	0	2	116	2	0	2
e0162	119	1	0	0	119	0	0	1	e0612	116	1	0	3	118	1	0	1
e0163	117	0	1	2	118	1	0	1	e0613	119	0	0	1	117	3	0	0
e0166	119	0	0	1	118	2	0	1	e0614	119	0	0	1	116	1	0	3
e0170	116	2	0	0	119	0	0	1	e0615	120	0	0	0	118	0	0	2
e0202	119	1	0	0	120	0	0	0	e0704	120	0	0	0	118	0	0	2
e0203	117	1	0	2	120	0	0	0	e0801	118	1	0	1	119	1	0	0
e0204	116	1	0	3	119	0	0	1	e0808	117	2	0	1	119	1	0	0
e0205	120	0	0	0	119	1	0	0	e0817	117	2	0	1	117	0	0	3
e0206	120	0	0	0	120	0	0	0	e0818	119	0	0	1	119	0	0	1
e0207	118	1	0	1	119	0	0	1	e1301	119	0	0	1	118	2	0	0
e0208	115	2	0	3	116	4	0	0	e1302	117	2	0	1	119	0	0	1
e0210	118	1	0	1	119	0	0	0	e1304	120	0	0	0	118	1	0	1

Table S2. Proposed scheme evaluation applied to MIT Fantasia database

Records	TP	TN	FP	FN
f1o01	120	0	0	0
f1o02	120	0	0	0
f1o03	114	1	3	2
f1o04	119	0	0	1
f1o05	120	0	0	0
f1o06	116	1	2	1
f1o07	118	1	0	1
f1o08	118	0	1	1
f1o09	120	0	0	0
f1o10	116	0	2	2
f1y01	118	1	1	0
f1y02	118	0	0	2
f1y03	119	0	0	1
f1y04	119	1	0	0
f1y05	120	0	0	0
f1y06	119	0	0	1
f1y07	120	0	0	0
f1y08	120	0	0	0
f1y09	120	0	0	0
f1y10	118	0	1	1
f2o01	120	0	0	0
f2o02	120	0	0	0
f2o03	116	1	3	0
f2o04	120	0	0	0
f2o05	115	1	3	1
f2o06	117	1	2	0
f2o07	118	1	0	1
f2o08	118	0	1	1
f2o09	120	0	0	0
f2o10	116	1	2	1
f2y01	119	0	1	0
f2y02	118	0	0	2
f2y03	116	0	1	3
f2y04	120	0	0	0
f2y05	120	0	0	0
f2y06	119	1	0	0
f2y07	119	0	1	0
f2y08	117	1	0	2
f2y09	117	0	2	1
f2y10	120	0	0	0

Table S3. Proposed scheme evaluation applied to collected ischemia database

Records	TP	TN	FP	FN
p1	30	0	0	0
p2	28	1	1	0
p3	28	1	1	0
p4	30	0	0	0
p5	28	1	0	1
p6	27	3	0	0
p7	30	0	0	0
p8	29	0	1	0
p9	29	1	0	0
p10	30	0	0	0
p11	30	0	0	0
p12	30	0	0	0
p13	30	0	0	0
p14	29	1	0	0
p15	29	1	0	0
p16	28	1	1	0
p17	29	0	1	0
p18	29	1	0	0
p19	30	0	0	0
p20	30	0	0	0
p21	29	0	1	0
p22	28	1	1	0
p23	27	2	0	1
p24	29	1	0	0
p25	29	1	0	0
p26	28	1	0	1
p27	30	0	0	0
p28	29	1	0	0
p29	29	0	1	0
p30	28	2	0	0
